# New Orleans Odontological Society

**Published:** 1884-03

**Authors:** Chas. Eckhardt

**Affiliations:** Secretary


### NEW ORLEANS ODONTOLOGICAL SOCIETY.

The regular monthly meeting of the New Orleans Odontological Society for November, was held at the office of Dr. W. S. Chandler, on the evening of the thirteenth, Dr. J. W. Adams presiding.

Nine members were present. Absent, Dr. P. J. Friedrichs.

The minutes of the previous meeting were read and approved.

Dr. C. Edmund Kells, Jr., read a paper entitled "Care of the Teeth," of which the following is a synopsis:

Dr. Kells first called attention to the great importance to dentists as well as to patients of the subject, "Care of the teeth," and opened with the statement that "Cleanliness is the bottom plank in the salvation of the teeth," and therefore every dentist should urge upon his patients the necessity for the proper use of the brush.

Not one in a thousand knows how to use the brush in a common-sense manner, and hence we often find deep furrows worn upon the labio-cervical margins of front teeth, attended with great recession of the gums, caused by a too violent and misguided use of the brush. Dr. Kells held that it was unnecessary to argue upon the bug theory, or the chemical theory, or the chemico-vital unification, but would trace back still further in the history of the process of disease, and call it want of cleanliness. He said that had he a sound and perfect tooth, he could keep it free from caries, provided he could polish each surface twice daily. He advised the daily use of floss silk, but could not depend much upon patients to use it regularly and faithfully. He also called attention to the many worthless patterns of tooth-brushes found in drug-stores and dental depots. He exhibited a sample brush of his own design, a curved brush, the back conforming to the dental arch, with bristles of an equal length, retaining thereby an even contour, short, and of medium stiffness. Dr. Kells claims that it is the only brush constructed upon a scientific basis, and showed samples of Dr. Palmer's, and other brushes, for comparison. He spoke of the manner of using the brush, and as few patients possess that knowledge, he keeps a set of continuous gum teeth with which to demonstrate to them how to use the brush.

He then spoke of dentifrices; objected to soap, if used exclusively; said it was a lubricant, and would glide over particles of calculus that a powder would remove; preferred a carbolized powder, or precipitated chalk, used once or twice daily; had no use for soaps, pastes, or creams. He advised mouth-washes, but merely as adjuncts; advised flushing by the auxiliary sanitary method. He concluded by recapitulating the main points of his essay: First—the brush; second—the dentifrice; third—the use of both. On these the salvation of the teeth depended.

DISCUSSION.

*Dr Bauer.*—Agrees with the sum and substance of the essay, except in the statement of the preservation of sound teeth, for he is not so sure of always saving a perfect tooth, because there are many conditions in which it may be lost, independently of caries.

*Dr. Kells.*—In reply, stated that he had reference to a tooth in which all conditions were favorable.

*Dr. Geo. J. Friedrichs.*—Thought Dr. Kells ought to have mentioned the tooth-pick, which was omitted in his essay. The brush, powders, etc., are all very well, but he holds that the tooth-pick is indispensable.

*Dr. Kells, Sr.*—Spoke of an instrument made of half-burnt clay, and shaped something like an anchor, which was used very effectively by Dr. Parmly in cleaning and polishing the teeth. He used it in connection with floss silk.

*Dr. Geo. J. Friedrichs.*—Said there is such a thing as abuse of the tooth-brush. Had often seen considerable abrasion of mucous membrane in mouths of patients who, just before coming to the office, wishing, no doubt, to convey the idea of great cleanliness, had indulged in a too vigorous and muscular use of the tooth-brush. Advised his patients to use precipitated chalk three times a week, to use the brush three times daily, after meals, and limited the time of brushing to fifteen seconds, when no harm could be done. Disapproved of the too vigorous use of the brush, and especially the ten to fifteen minute exercise, as is customary with some people. Never saw a tooth lost from over-brushing.

*Dr. Chas. E. Kells.*—The teeth should not be brushed on first rising in the morning, but should be subjected to a thorough rinsing with plain water, in order to dislodge and carry off the viscid mucous secretions; but he advised a thorough brushing with powder after breakfast.

*Dr. Bauer.*—If necessary to choose, would sooner do without washing his face than to omit brushing his teeth; thought that before retiring at night was the most important time to clean the teeth, as it was an accepted theory that they decay more at night than during the day.

*Dr. Chas. E. Kells.*—Has a lady patient, whose teeth, despite all his and her care, will decay; she now uses, in connection with other cleansing appliances, a syringe for her teeth, in order to more thoroughly effect the cleansing process; has other patients who have splendid teeth, but who bestow upon them comparatively little care.

*Dr. Geo. J. Friedrichs.*—The same principle holds good with people who do not use a brush; their teeth are often found without caries. Chewing of tobacco is a preservative of the teeth. It stimulates the flow of saliva, keeps the fluids of the mouth in constant motion by the movements of the tongue, lips and cheeks, thereby constantly cleansing the masticatory organs. Tobacco is used so excessively by some as to grind away the teeth very rapidly, thus causing inflammation of the gingival portion of the gum around the teeth. He spoke of the green stain on the teeth of persons who brush their teeth, and described a method of removing it.

*Dr. Viet.*—Thought that the green stain was due to negligence had noticed it mostly upon teeth of children; said the stain would disappear after several washings.

The discussion here became general, and was participated in by Drs. Kells, Jr., W. S. Chandler, and others.

INCIDENTS OF OFFICE PRACTICE.

Dr. A. G. Friedrichs related the case of a gentleman in the drug department of Touro Infirmary, who, suffering extremely, came to him for relief. Upon examination he found the first upper bicuspid and lower cuspid roots the source of trouble; extracted both, and patient departed apparently happy. Afterwards, upon inquiry, he was told by the patient that Dr. Godfrey relieved his pain by lancing an abscess in his ear.

On motion of Dr. Geo. J. Friedrichs, the society adjourned to meet Tuesday, December 11, 1883.

CHAS. ECKHARDT, D. D. S., *Secretary.*

Officers of New Orleans Odontological Society:

Dr. J. W. ADAMS, President.

Dr. P. J. FRIEDRICHS, Vice-President.

Dr. CHAS. ECKHARDT, Secretary and Treasurer.

